# Equivalence and non inferiority trials

**DOI:** 10.1186/1745-6215-12-S1-A28

**Published:** 2011-12-13

**Authors:** Ralph B D’Agostino

## 

Many effective drugs, biologics and devices exist. Because of this, it is often considered unethical to undertake placebo controlled clinical trials to evaluate new treatments. Rather, randomized active control non-inferiority trials have become the norm. These trials, however, are accompanied by serious issues including selecting an active comparator, identifying previously run active comparator placebo controlled trials, deciding upon the form of comparison (e.g., differences or ratios), setting a non-inferiority margin, designing a new study where consistency with past trials hold, finding study sites where the trial can be performed, selecting the appropriate analysis set (Intent-to-treat or per protocol) and determining sample size. These issues have proven to be more problematic than naively anticipated. Further with the accumulation and advancement of these studies and the attempt to expand their use, new problems arose and others can be anticipated to arise. Some common problems are the realization that consistency with previous active comparator placebo trials does not hold, that the multiple available ad hoc (non-theory driven) tests present confusion rather than help and that the outcome data for the active comparator often do not match the anticipated results. Newer problems are the attempts of switching from non-inferiority to superiority testing and superiority to non-inferiority testing, the use of interim analyses, multiple treatments and dealing with recurrent events as the trial outcomes. In this talk we present a brief overview of the above beginning with the migration from equivalency test to non-inferiority test, moving through the issues and with emphasis on the major problems.

